# A case of solitary neurofibroma in the maxillary gingiva

**DOI:** 10.1093/jscr/rjac222

**Published:** 2022-07-30

**Authors:** Yuko Komatsu, Yasunori Takeda, Tadashi Kawai, Shunichi Sasou, Kazuaki Takahashi, Hiroyuki Yamada, Shu Ishibashi

**Affiliations:** Division of Oral and Maxillofacial Surgery, Department of Oral and Maxillofacial Reconstructive Surgery, School of Dentistry, Iwate Medical University, Morioka, Iwate, Japan; Department of Oral Surgery, Hachinohe Red Cross Hospital, Hachinohe, Aomori, Japan; Division of Clinical Pathology, Department of Oral and Maxillofacial Reconstructive Surgery, School of Dentistry, Iwate Medical University, Shiwa-gun, Iwate, Japan; Division of Oral and Maxillofacial Surgery, Department of Oral and Maxillofacial Reconstructive Surgery, School of Dentistry, Iwate Medical University, Morioka, Iwate, Japan; Department of Pathology, Hachinohe Red Cross Hospital, Hachinohe, Aomori, Japan; Department of Oral Surgery, Hachinohe Red Cross Hospital, Hachinohe, Aomori, Japan; Division of Oral and Maxillofacial Surgery, Department of Oral and Maxillofacial Reconstructive Surgery, School of Dentistry, Iwate Medical University, Morioka, Iwate, Japan; Department of Oral Surgery, Hachinohe Red Cross Hospital, Hachinohe, Aomori, Japan

**Keywords:** neurofibroma, gingiva, solitary

## Abstract

Neurofibromas are benign tumors. They are known to be a manifestation of von Recklinghausen’s disease (neurofibromatosis type 1) and tend to be generalized and rarely solitary. In this report, we present a case of solitary neurofibroma in the maxillary gingiva. A 39-year-old woman presented with a chief complaint of swollen gingiva. There were no special findings in her medical or family history. After a biopsy was performed under local anesthesia and a diagnosis of neurofibroma was made, tumor resection was performed under general anesthesia. The patient’s progress was good, with no recurrence.

## INTRODUCTION

Neurofibromas are benign tumors. The 2017 World Health Organization classification defines it as a benign peripheral nerve sheath tumor composed of a mixture of palisade cells, neuropericytes, fibroblasts and axons [1]. Neurofibromas are known to be a manifestation of von Recklinghausen’s disease (neurofibromatosis type 1) and tend to be generalized and rarely solitary. Neurofibromatosis type 1, together with neurofibromatosis type 2 and schwannomatosis, constitutes a group of neurofibromatoses, each with an incidence of 1 in 3000 births [2]. Currently, neurofibromatosis type 1 is known to be caused by heterozygous mutations in the NF1 gene, but due to the diversity of genotypes and phenotypes, the overall correlation between genotype and phenotype is not known [3]. In this study, we report a case of solitary neurofibroma in the maxillary gingiva.

## CASE REPORT

**Figure 1:**
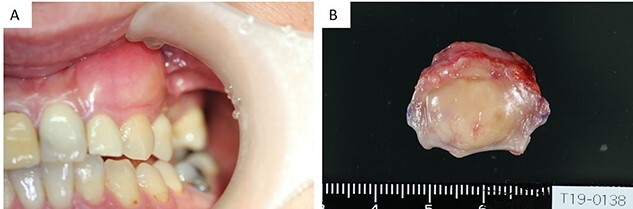
(**A**) Intraoral examination showing a bulge in the buccal gingiva of the left maxillary canine. (**B**) Intraoperative findings: a pale yellow explant covered with a membrane.

A 39-year-old woman presented with a chief complaint of swollen left maxillary gingiva. She had been aware of the swollen gingiva for 6 years but had neglected it because it was not painful. Her family dentist recommended a thorough examination, and she visited our department for her first visit. There were no special findings in her medical or family history. The extraoral examination was normal, but intraoral examination revealed a bulge in the buccal gingiva of the left maxillary canine (Fig. 1A). The mucosa in the bulge area was normal and hard like bone.

**Figure 2:**
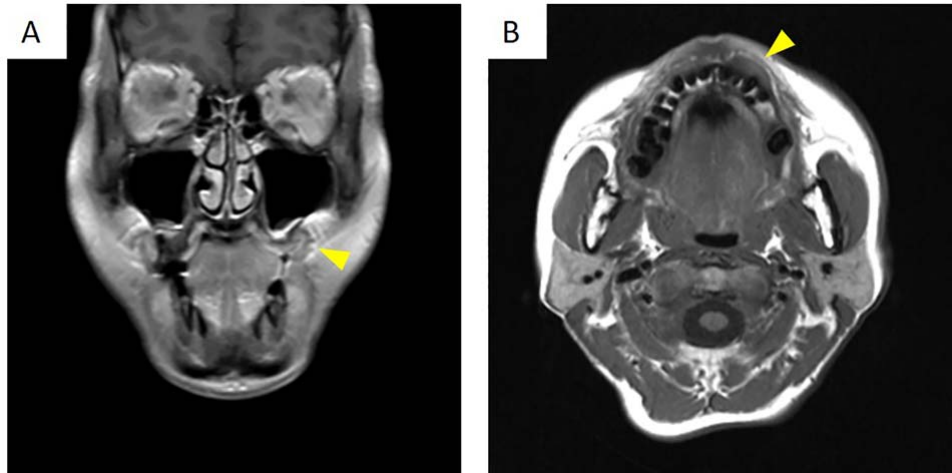
On magnetic resonance imaging, contrast-enhanced T1-weighted images showed a row signal. (**A**) Frontal cross-section. (**B**) Horizontal cross-section. The arrowheads indicate the tumor.

There was no evidence of bony swelling or resorption on computed tomography (data not shown). On magnetic resonance imaging, contrast-enhanced T1-weighted images showed a mass with low signal (Fig. 2A and B), measuring 19.3 × 12.1 × 9.2 (length × breadth × thickness) mm. It showed thickening of the mucosa at the left upper gingival cheek transition. The mass did not infiltrate the alveolar bone and had a relatively clear border with the surrounding area.

**Figure 3:**
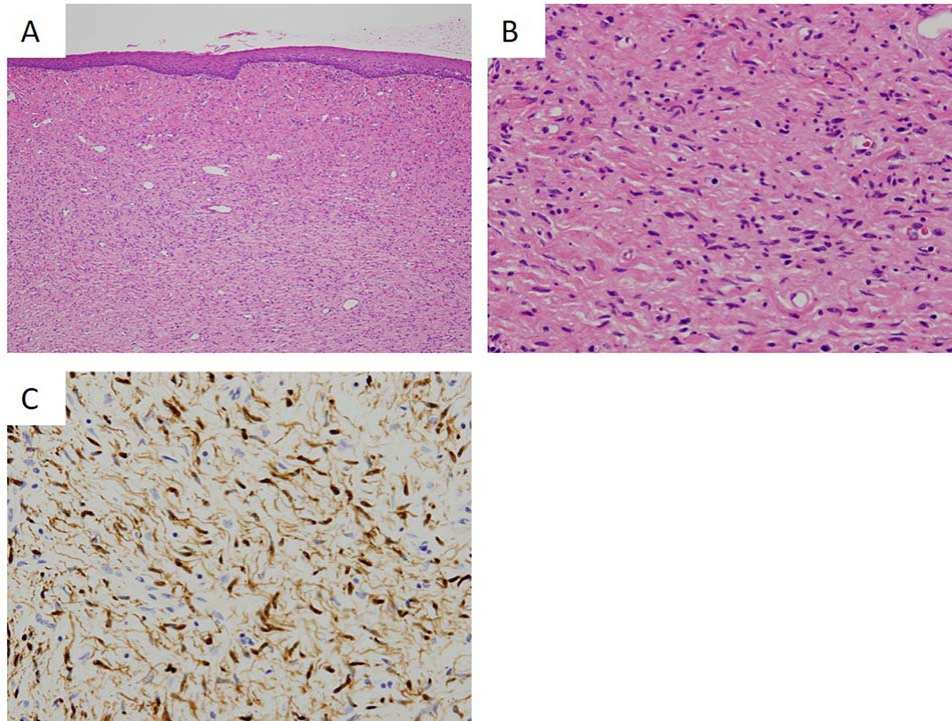
Histopathological findings of the tumor. (**A**) The lesion is a poorly circumscribed tumor in the submucosal area (hematoxylin and eosin (HE), ×40). (**B**) Proliferation of elongated and slender cells with round, ovoid and comma-shaped nuclei in a background of sinuous collagen fibers and myxoid matrix (HE, ×200). (**C**) Immunohistochemical staining of tumor cells showing positivity for S100 (S100, ×200).

The specimens were collected from the gingival cheek transition area. The gingival mucosa was normal and contained a yellow mass that was full, elastic and hard. The specimen was a yellowish-white, substantial, inelastic mass. At the time of biopsy, the histopathological diagnosis was neurofibroma.

**Table 1:** Solitary neurofibromas of the gingiva have been reported between 2000 and 2021

Year	Author	Age	Gender	Location	Follow-up-period	Recurrence
2002	Shimoyama [10]	25	Female	Maxillary anterior	2 years	-
2008	Gosavi [6]	17	Female	Maxillary anterior		-
2009	Depprich [11]	64	Female	Mandibular posterior	1 year	-
2010	Ohno [8]	32	Female	Mandibular posterior	5 years	-
2013	Gosavi [6]	10	Female	Maxillary posterior		-
2013	Gosavi [6]	18	Male	Mandibular posterior		-
2013	Suramya [7]	57	Female	Maxillary anterior	1 year	-
2013	Pawar [9]	25	Female	Maxillary posterior	1 year	-
2013	Gosavi [6]	14	Female	Maxillary anterior		+
2014	Gosavi [6]	56	Male	Mandibular lingual		-
2014	Dayal [12]	72	Male	detail unknown	15 years	-
2016	Gosavi [6]	35	Female	Maxillary posterior		-
2016	George [13]	22	Female	Mandibular posterior	1.5 years	-
2018	Gosavi [6]	30	Male	Maxillary anterior		-
2018	Gosavi [6]	47	Male	Mandibular buccal		-
2022	Our case	39	Female	Maxillary anterior	1 year	-

Since there was no other sign of neurofibromatosis, a solitary neurofibroma was diagnosed. The patient underwent tumor resection under general anesthesia. The resection included the subperiosteum, with a surgical margin of 5 mm. The surrounding bone was smooth, stained with pyoctanine blue solution and one layer was removed. An artificial dermis was applied to the raw surface. The explant was pale yellow and covered with a membrane (Fig. 1B). The patient was followed up for 1 year after surgery and showed no evidence of recurrence.

The lesion was a poorly circumscribed tumor proliferating in the submucosal area (Fig. 3A). The tumor was composed of elongated and slender cells with round, ovoid and comma-shaped nuclei in a background of sinuous collagen fibers and myxoid matrix (Fig. 3B). Neither cellular atypia nor mitotic figures were observed. Immunohistochemically, the nucleus and cytoplasm of these tumor cells were positive for S100 (Fig. 3C) but negative for neuron-specific enolase.

## DISCUSSION

Neurofibromatosis type 1 is a hereditary neurocutaneous disease with significant effects on the nervous system, eyes, skin and bones [4]. The clinical manifestations of neurofibromatosis type 1 include neurofibromas, café-au-lait spots, freckles on the skin, skeletal dysplasia, Lisch nodules and optic gliomas [5]. Oral neurofibromas are most commonly found on the tongue and rarely on the gingiva, jawbone or floor of the mouth [6]. Neurofibromatosis type 1 is a widely known disease, but there is still a lack of information on solitary neurofibromas. To the best of our knowledge, only 16 cases of solitary neurofibromas of the gingiva have been reported between 2000 and 2021, including our case [7–13]. The 16 cases are summarized in Table 1. The patients’ ages ranged from 10 to 72 years, with a median age of 31 years. The disease was more common in females, with 5 male cases and 11 female cases. There were nine maxillary cases, six mandibular cases and one case with no details. In the maxillary cases, six involved the anterior region and three involved the posterior region, with the anterior region being the most common. Information on the presence or absence of tumor membranes was available in only 3 of the 16 cases, with 2 cases showing no membranes and 1 case showing a membrane. The number of reports was too small to describe the relationship with recurrence or surgical technique. There was only one case of recurrence [5]; however, whether a membrane was present was not reported, nor were the details about surgical techniques. In our case, a surgical margin was established, and the surrounding bone was removed from one layer. Further, the patient’s progress was good, with no recurrence after surgery. In addition, one case of subcutaneous neurofibroma with features of malignant peripheral nerve sheath tumor transformation has been reported in the field of dermatology but not in the head and neck region [14].

No racial differences have been reported for neurofibromatosis type 1, but 14 of 16 cases we collected were reported from Asian countries, suggesting a regional or racial predilection in solitary neurofibroma. In addition, the diagnosis of solitary neurofibroma requires a clinical rejection of neurofibromatosis type 1. However, it is difficult to rule out neurofibromatosis type 1, especially in young people because some of neurofibromatosis type 1 are known to occur without obvious genetic mutations or familial effects, and the main symptoms may occur at different times [3, 5]. This may be the reason why the cases of solitary neurofibromas are rarely reported. Neurofibromas are not lesions with a high recurrence rate, but even solitary lesions should continue to be followed because findings consistent with neurofibromatosis type 1 may later appear.
